# Sharp Cheeger–Buser Type Inequalities in $\mathsf {RCD}(K,\infty )$ Spaces

**DOI:** 10.1007/s12220-020-00358-6

**Published:** 2020-02-14

**Authors:** Nicolò De Ponti, Andrea Mondino

**Affiliations:** 1 Dipartimento di Matematica “Casorati”, Università degli Studi di Pavia, Pavia, Italy; 2 Mathematical Institute, University of Oxford, Oxford, UK

**Keywords:** Ricci curvature, Metric measure spaces, First eigenvalue laplace operator, Cheeger inequality, Buser inequality

## Abstract

The goal of the paper is to sharpen and generalise bounds involving Cheeger’s isoperimetric constant *h* and the first eigenvalue $\lambda _{1}$ of the Laplacian. A celebrated lower bound of $\lambda _{1}$ in terms of *h*, $\lambda _{1}\ge h^{2}/4$, was proved by Cheeger in 1970 for smooth Riemannian manifolds. An upper bound on $\lambda _{1}$ in terms of *h* was established by Buser in 1982 (with dimensional constants) and improved (to a dimension-free estimate) by Ledoux in 2004 for smooth Riemannian manifolds with Ricci curvature bounded below. The goal of the paper is twofold. First: we sharpen the inequalities obtained by Buser and Ledoux obtaining a dimension-free sharp Buser inequality for spaces with (Bakry–Émery weighted) Ricci curvature bounded below by $K\in \mathbb R$ (the inequality is sharp for $K>0$ as equality is obtained on the Gaussian space). Second: all of our results hold in the higher generality of (possibly non-smooth) metric measure spaces with Ricci curvature bounded below in synthetic sense, the so-called $\mathsf {RCD}(K,\infty )$ spaces.

## Introduction

Throughout the paper $(X, \mathsf {d})$ will be a complete metric space and $\mathfrak m$ will be a non-negative Borel measure on *X*, finite on bounded subsets. The triple $(X, \mathsf {d}, \mathfrak m)$ is called *metric measure space*, m.m.s. for short. We denote by $\mathsf {Lip}(X)$ the space of real-valued Lipschitz functions over *X* and we write $f\in \mathsf {Lip}_b(X)$ if $f\in \mathsf {Lip}(X)$ and *f* is bounded with bounded support. Given $f\in \mathsf {Lip}(X)$ its slope $|\nabla f|(x)$ at $x\in X$ is defined by

1 $$\begin{aligned} |\nabla f|(x):=\limsup _{y\rightarrow x} \frac{|f(y)-f(x)|}{\mathsf {d}(y,x)}, \end{aligned}$$

with the convention $|\nabla f|(x)=0$ if *x* is an isolated point. The first non-trivial eigenvalue of the Laplacian is characterized as follows:

- If $\mathfrak m(X)<\infty$, the non-zero constant functions are in $L^2(X,\mathfrak m)$ and are eigenfunctions of the Laplacian with eigenvalue 0. In this case, we set
  2 $$\begin{aligned} \lambda _1= \inf \bigg \{\frac{\int _X |\nabla f|^2 d\mathfrak m}{\int _X |f|^2d\mathfrak m}: \ 0\not \equiv f\in \mathsf {Lip}_{b}(X), \int _X f\, d\mathfrak m=0\bigg \}. \end{aligned}$$
- When $\mathfrak m(X)=\infty$, 0 may not be an eigenvalue of the Laplacian. Thus, we set
  3 $$\begin{aligned} \lambda _0= \inf \bigg \{\frac{\int _X |\nabla f|^2 d\mathfrak m}{\int _X |f|^2d\mathfrak m}: \ \ 0\not \equiv f\in \mathsf {Lip}_b(X) \bigg \}. \end{aligned}$$

At this level of generality, the spectrum of the Laplacian may not be discrete (see Remark 1.3 for more details); in any case the definitions (2) and (3) make sense, and one can investigate bounds on $\lambda _{1}$ and $\lambda _{0}$.

Note that $\lambda _0$ may be zero (for instance if $\mathfrak m(X)<\infty$ or if $(X,\mathsf {d},\mathfrak m)$ is the Euclidean space $\mathbb {R}^d$ with the Lebesgue measure) but there are examples when $\lambda _0> 0$: for instance in the Hyperbolic plane $\lambda _{0}=1/4$ and more generally on an *n*-dimensional simply connected Riemannian manifold with sectional curvatures bounded above by $k<0$ it holds $\lambda _{0}\ge (n-1)^{2} |k| /4$ (see [27]).

Given a Borel subset $A\subset X$ with $\mathfrak m(A)<\infty$, the *perimeter* $\mathrm {Per}(A)$ is defined as follows (see for instance [28]):

$$\begin{aligned} \mathrm {Per}(A):=\inf \bigg \{\liminf _{n\rightarrow \infty }\int _X |\nabla f_n|d\mathfrak m: f_n\in \mathsf {Lip}_b(X), f_n\rightarrow \chi _A \ \mathrm {in} \ L^1(X,\mathfrak m)\bigg \}. \end{aligned}$$

In 1970, Cheeger [17] introduced an isoperimetric constant, now known as *Cheeger constant*, to bound from below the first eigenvalue of the Laplacian. The *Cheeger constant* of the metric measure space $(X,\mathsf {d},\mathfrak m)$ is defined by

4 $$\begin{aligned} h(X):= {\left\{ \begin{array}{ll} \inf \left\{ \frac{\mathrm {Per}(A)}{\mathfrak m(A)}\, :\, A\subset X \text { Borel subset with }\mathfrak m(A)\le \mathfrak m(X)/2 \right\} &{} \text{ if } \mathfrak m(X)<\infty \\ \inf \left\{ \frac{\mathrm {Per}(A)}{\mathfrak m(A)}\, :\, A\subset X \text { Borel subset with }\mathfrak m(A)<\infty \right\} &{}\text{ if } \mathfrak m(X)=\infty . \end{array}\right. } \end{aligned}$$

The lower bound obtained in [17] for compact Riemannian manifolds, now known as *Cheeger inequality*, reads as

5 $$\begin{aligned} \lambda _{1}\ge \frac{1}{4} h(X)^{2}. \end{aligned}$$

As proved by Buser [11], the constant 1/4 in (5) is optimal in the following sense: for any $h > 0$ and $\varepsilon >0$, there exists a closed (i.e. compact without boundary) two-dimensional Riemannian manifold (*M*, *g*) with $h(M)=h$ and such that $\lambda _{1}\le \frac{1}{4} h(M)^{2}+\varepsilon$.

The paper [17] is in the framework of smooth Riemannian manifolds; however, the stream of arguments (with some care) extends to general metric measure spaces. For the reader’s convenience, we give a self-contained proof of (5) for m.m.s. in the Appendix (see Theorem 3.6).

Cheeger’s inequality (5) revealed to be extremely useful in proving lower bounds on the first eigenvalue of the Laplacian in terms of the isoperimetric constant *h*. It was thus an important discovery by Buser [12] that also an upper bound for $\lambda _{1}$ in terms of *h* holds, where the inequality explicitly depends on the lower bound on the Ricci curvature of the smooth Riemannian manifold. More precisely, Buser [12] proved that for any compact Riemannian manifold of dimension *n* and $\mathrm{Ric}\ge K$, $K\le 0$ it holds

6 $$\begin{aligned} \lambda _1\le 2\sqrt{-(n-1)K}h+10h^2. \end{aligned}$$

Note that the constant here is dimension-dependent. For a complete connected Riemannian manifold with $\mathrm{Ric}\ge K$, $K\le 0$, Ledoux [24] remarkably showed that the constant can be chosen to be independent of the dimension:

7 $$\begin{aligned} \lambda _1\le \max \{6\sqrt{-K}h,36h^2\}. \end{aligned}$$

The goal of the present work is twofold:

1. The main results of the paper (Theorem 1.1 and Corollary 1.2) improve the constants in both the Buser-type inequalities (6)-(7) in a way that now the inequality is sharp for $K>0$ (as equality is attained on the Gaussian space).
2. The inequalities are established in the higher generality of (possibly non-smooth) metric measure spaces satisfying Ricci curvature lower bounds in synthetic sense, the so-called $\mathsf {RCD}(K,\infty )$ spaces.

For the precise definition of $\mathsf {RCD}(K,\infty )$ space, we refer the reader to Section 2. Here, let us just recall that the $\mathsf {RCD}(K,\infty )$ condition was introduced by Ambrosio-Gigli-Savaré [6] (see also [4]) as a refinement of the $\mathsf {CD}(K,\infty )$ condition of Lott-Villani [26] and Sturm [33]. Roughly, a $\mathsf {CD}(K,\infty )$ space is a (possibly infinite-dimensional, possibly non-smooth) metric measure space with Ricci curvature bounded from below by *K*, in a synthetic sense. While the $\mathsf {CD}(K,\infty )$ condition allows Finsler structures, the main point of $\mathsf {RCD}$ is to reinforce the axiomatization (by asking linearity of the heat flow) to rule out Finsler structures and thus isolate the “possibly non-smooth Riemannian structures with Ricci curvature bounded below”. It is out of the scopes of this introduction to survey the long list of achievements and results proved for $\mathsf {CD}$ and $\mathsf {RCD}$ spaces (to this aim, see the Bourbaki seminar [34] and the recent ICM-Proceeding [1]). Let us just mention that a key property of both $\mathsf {CD}$ and $\mathsf {RCD}$ is the stability under measured Gromov–Hausdorff convergence (or more generally ${\mathbb D}$-convergence of Sturm [6, 33], or even more generally pointed measured Gromov convergence [20]) of metric measure spaces. In particular pointed measured Gromov–Hausdorff limits of Riemannian manifolds with Ricci bounded below, the so-called *Ricci limits*, are examples of (possibly non-smooth) $\mathsf {RCD}$ spaces. Let us also recall that weighted Riemannian manifolds with Bakry-Émery Ricci tensor bounded below are also examples of $\mathsf {RCD}$ spaces; for instance the Gaussian space $(\mathbb R^{d}, |\cdot |, (2\pi )^{-d/2} e^{-|x|^{2}/2} d\mathcal L^{d}(x))$, $1\le d\in \mathbb {N}$, satisfies $\mathsf {RCD}(1,\infty )$. It is also worth recalling that if $(X,\mathsf {d},\mathfrak m)$ is an $\mathsf {RCD}(K,\infty )$ space for some $K>0$, then $\mathfrak m(X)<\infty$; since scaling the measure by a constant does not affect the synthetic Ricci curvature lower bounds, when $K>0$, without loss of generality one can then assume $\mathfrak m(X)=1$.

To state our main result, it is convenient to set

8 $$\begin{aligned} J_K(t)={\left\{ \begin{array}{ll}\sqrt{\frac{2}{\pi K}}\arctan \Big (\sqrt{e^{2Kt}-1}\Big ) \ \ &{}\text {if} \ \ K>0,\\ \frac{2}{\sqrt{\pi }}\sqrt{t} \ \ &{}\text {if} \ \ K=0,\\ \sqrt{-\frac{2}{\pi K}}{{\,\mathrm{arctanh}\,}}{\Big (\sqrt{1-e^{2Kt}}\Big )} \ \ &{}\text {if} \ \ K<0.\end{array}\right. } \qquad \forall t>0 \end{aligned}$$

The aim of the paper is to prove the following theorem.

### Theorem 1.1

(Sharp implicit Buser-type inequality for $\mathsf {RCD}(K,\infty )$ spaces) Let $(X,\mathsf {d},\mathfrak m)$ be an $\mathsf {RCD}(K,\infty )$ space, for some $K\in \mathbb {R}$.

- In case $\mathfrak m(X)=1$, then
  9 $$\begin{aligned} h(X)\ge \sup _{t>0} \frac{1-e^{-\lambda _1t}}{J_K(t)}. \end{aligned}$$
  The inequality is sharp for $K>0$, as equality is achieved for the Gaussian space $(\mathbb R^{d}, |\cdot |, (2\pi )^{-d/2} e^{-|x|^{2}/2} d{\mathcal {L}}^{d}(x))$, $1\le d\in \mathbb {N}$.
- In case $\mathfrak m(X)=\infty$, then
  10 $$\begin{aligned} h(X)\ge 2\sup _{t>0} \frac{1-e^{-\lambda _0t}}{J_K(t)}. \end{aligned}$$

Using the expression (8) of $J_{K}$, in the next corollary we obtain more explicit bounds.

### Corollary 1.2

(Explicit Buser inequality for $\mathsf {RCD}(K,\infty )$ spaces) Let $(X,\mathsf {d},\mathfrak m)$ be an $\mathsf {RCD}(K,\infty )$ space, for some $K\in \mathbb {R}$.

- Case $K>0$. If $\frac{K}{\lambda _{1}}\ge c>0$, then
  11 $$\begin{aligned} \lambda _{1}\le \frac{\pi }{2c} h(X)^{2}. \end{aligned}$$
  The estimate is sharp, as equality is attained on the Gaussian space $(\mathbb R^{d}, |\cdot |, (2\pi )^{-d/2} e^{-|x|^{2}/2} d\mathcal L^{d}(x))$, $1\le d\in \mathbb {N}$, for which $K=1, \lambda _{1}=1, h(X)= (2/\pi )^{1/2}$.
- Case $K=0$, $\mathfrak m(X)=1$. It holds
  12 $$\begin{aligned} \lambda _{1}\; \le \; \frac{4}{\pi } h(X)^2 \inf _{T>0} \frac{T}{(1-e^{-T})^{2}} \; < \; \pi h(X)^2. \end{aligned}$$
  In case $\mathfrak m(X)=\infty$, the estimate (12) holds replacing $\lambda _{1}$ with $\lambda _{0}$ and *h*(*X*) with *h*(*X*)/2.
- Case $K<0$, $\mathfrak m(X)=1$. It holds
  13 $$\begin{aligned} \lambda _1&\le \max \bigg \{\sqrt{-K}\frac{\sqrt{2}\log \big (e+\sqrt{e^{2}-1}\big )}{\sqrt{\pi }(1-\frac{1}{e})}h(X),\frac{2\Big (\log \big (e+\sqrt{e^{2}-1}\big )\Big )^2}{\pi \Big (1-\frac{1}{e}\Big )^2}h(X)^2\bigg \} \nonumber \\&<\max \left\{ \frac{21}{10} \sqrt{-K}h(X),\frac{22}{5}h(X)^2 \right\} . \end{aligned}$$
  In case $\mathfrak m(X)=\infty$, the estimate (13) holds replacing $\lambda _{1}$ with $\lambda _{0}$ and *h*(*X*) with *h*(*X*)/2.

### Remark 1.3

Even if the definitions of $\lambda _{0}$ and $\lambda _{1}$ as in (2) and (3) make sense regardless of the discreteness of the spectrum of the Laplacian (as well as the proofs of the above results), it is worth to mention some cases of interest where the Laplacian has discrete spectrum.

It was proved in [20] that an $\mathsf {RCD}(K,\infty )$ space, with $K>0$ (or with finite diameter) has discrete spectrum (as the Sobolev imbedding $\mathbb {V}$ into $L^{2}$ is compact). Even in case of infinite measure the embedding of $\mathbb {V}$ in $L^2$ may be compact. An example is given by $\mathbb {R}$ with the Euclidean distance $\mathsf {d}(x,y)=|x-y|$ and the measure $\mathfrak m:=\frac{1}{\sqrt{2\pi }}e^{x^2/2}d\mathcal {L}^1.$ It is a $\mathsf {RCD}(-1,\infty )$ space and a result of Wang [35] ensures that the spectrum is discrete.

### Comparison with Previous Results in the Literature

Theorem 1.1 and Corollary 1.2 improve the known results about Buser-type inequalities in several aspects. First of all, the best results obtained before this paper are the aforementioned estimates (6)-(7) due to Buser [12] and Ledoux [24] for smooth complete Riemannian manifolds satisfying $\mathrm{Ric}\ge K$, $K\le 0$. Let us stress that the constants in Corollary 1.2 improve the ones in both (6)-(7) and are dimension-free as well. In addition, the improvements of the present paper are:

- In case $K>0$, the inequalities (9) and (11) are sharp (as equality is attained on the Gaussian space).
- The results hold in the higher generality of (possibly non-smooth) $\mathsf {RCD}(K,\infty )$ spaces.

The proof of Theorem 1.1 is inspired by the semi-group approach of Ledoux [23, 24], but it improves upon using Proposition 3.1 in place of:

- A dimension-dependent Li-Yau inequality, in [23].
- A weaker version of Proposition 3.1 (see [24, Lemma 5.1]) analyzed only in case $K\le 0$, in [24].

Theorem 1.1 and Corollary 1.2 are also the first *upper bounds* in the literature of $\mathsf {RCD}$ spaces for the first eigenvalue of the Laplacian. On the other hand, *lower bounds* on the first eigenvalue of the Laplacian have been thoroughly analyzed in both $\mathsf {CD}$ and $\mathsf {RCD}$ spaces: the sharp Lichnerowitz spectral gap $\lambda _{1}\ge KN/(N-1)$ was proved under the (non-branching) $\mathsf {CD}(K,N)$ condition by Lott-Villani [25], under the $\mathsf {RCD}^{*}(K,N)$ condition by Erbar-Kuwada-Sturm [18], and generalized by Cavalletti and Mondino [14] to a sharp spectral gap for the *p*-Laplacian for essentially non-branching $\mathsf {CD}^{*}(K,N)$ spaces involving also an upper bound on the diameter (together with rigidity and almost rigidity statements). Jiang-Zhang [21] independently showed, for $p=2$, that the improved version under an upper diameter bound holds for $\mathsf {RCD}^*(K,N)$. The rigidity of the Lichnerowitz spectral gap for $\mathsf {RCD}^*(K,N)$ spaces, $K>0$, $N\in (1,\infty )$, known as Obata’s Theorem was first proved by Ketterer [22]. The rigidity in the Lichnerowitz spectral gap for $\mathsf {RCD}(K,\infty )$ spaces, $K>0$, was recently proved by Gigli-Ketterer-Kuwada-Ohta [19]. Local Poincaré inequalities in the framework of $\mathsf {CD}(K,N)$ and $\mathsf {CD}(K,\infty )$ spaces were proved by Rajala [30]. Finally various lower bounds, together with rigidity and almost rigidity statements for the *Dirichlet first eigenvalue* of the Laplacian, have been proved by Mondino-Semola [29] in the framework of $\mathsf {CD}$ and $\mathsf {RCD}$ spaces. Lower bounds on Cheeger’s isoperimetric constant have been obtained for (essentially non-branching) $\mathsf {CD}^*(K,N)$ spaces by Cavalletti-Mondino [13–15] and for $\mathsf {RCD}(K,\infty )$ spaces ($K>0$) by Ambrosio-Mondino [7]. The local and global stability properties of eigenvalues and eigenfunctions in the framework of $\mathsf {RCD}$ spaces have been investigated by Gigli-Mondino-Savaré in [20], and by Ambrosio-Honda in [2, 3].

## Preliminaries

Throughout the paper, unless otherwise stated, we assume $(X,\mathsf {d})$ is a complete and separable metric space. We endow $(X,\mathsf {d})$ with a reference $\sigma$-finite non-negative measure $\mathfrak m$ over the Borel $\sigma$-algebra $\mathcal {B}$, with $\textsf {supp}(\mathfrak m)=X$ and satisfying an exponential growth condition, namely that there exist $x_0\in X$, $M>0$ and $c\ge 0$ such that

$$\begin{aligned} \mathfrak m(B_r(x_0))\le M\exp (cr^2) \ \ \text {for every} \ r\ge 0. \end{aligned}$$

Possibly enlarging $\mathcal {B}$ and extending $\mathfrak m$, we assume that $\mathcal {B}$ is $\mathfrak m$-complete. The triple $(X,\mathsf {d},\mathfrak m)$ is called metric measure space, m.m.s for short.

We denote by $\mathcal {P}_2(X)$ the space of probability measures on *X* with finite second moment and we endow this space with the Kantorovich–Wasserstein distance $W_2$ defined as follows: for $\mu _0,\mu _1 \in \mathcal {P}_{2}(X)$ we set

14 $$\begin{aligned} W_2^2(\mu _0,\mu _1) := \inf _{ \pi } \int _{X\times X} \mathsf {d}^2(x,y) \, d\pi , \end{aligned}$$

where the infimum is taken over all $\pi \in \mathcal {P}(X \times X)$ with $\mu _0$ and $\mu _1$ as the first and the second marginal.

The *relative entropy functional* $\mathsf {Ent}_{\mathfrak m}:\mathcal {P}_2(X)\rightarrow \mathbb R\cup \{\infty \}$ is defined as

15 $$\begin{aligned} \mathsf {Ent}_{\mathfrak m}(\mu ):={\left\{ \begin{array}{ll} \int \rho \log \rho \, d\mathfrak m\ &{}\text {if} \ \mu =\rho \mathfrak m, \\ \infty \ &{}\text {otherwise}.\end{array}\right. } \end{aligned}$$

A curve $\gamma :[0,1]\rightarrow X$ is a *geodesic* if

16 $$\begin{aligned} \mathsf {d}(\gamma _s,\gamma _t)= |t-s|\, \mathsf {d}(\gamma _0,\gamma _1) \ \ \ \ \ \forall s,t\in [0,1]. \end{aligned}$$

In the sequel we use the notation:

$$\begin{aligned} D(\mathsf {Ent}_{\mathfrak m}):=\{\mu \in \mathcal {P}_2(X) \,:\, \mathsf {Ent}_{\mathfrak m}(\mu )\in \mathbb R\}. \end{aligned}$$

We now define the $\mathsf {CD}(K,\infty )$ condition, coming from the seminal works of Lott-Villani [26] and Sturm [33].

### Definition 2.1

($\mathsf {CD}(K,\infty )$ condition) Let $K\in \mathbb {R}$. We say that $(X,\mathsf {d},\mathfrak m)$ is a $\mathsf {CD}(K,\infty )$ space provided that for any $\mu ^0,\mu ^1\in D(\mathsf {Ent}_{\mathfrak m})$ there exists a $W_2$-geodesic $(\mu _t)$ such that $\mu _0=\mu ^0$, $\mu _1=\mu ^1$ and

17 $$\begin{aligned} \mathsf {Ent}_{\mathfrak m}(\mu _t)\le (1-t)\mathsf {Ent}_{\mathfrak m}(\mu _0)+t\mathsf {Ent}_{\mathfrak m}(\mu _1)-\frac{K}{2}t(1-t)W_2^2(\mu _0,\mu _1). \end{aligned}$$

The space of continuous function $f:X\rightarrow \mathbb R$ is denoted by $\mathcal {C}(X)$ and the Lebesgue space by $L^p(X,\mathfrak m)$, $1\le p\le \infty$.

The *Cheeger energy* (introduced in [16] and further studied in [5]) is defined as the $L^{2}$-lower semicontinuous envelope of the functional $f \mapsto \frac{1}{2}\int _X |\nabla f|^2d\mathfrak m$, i.e.:

18 $$\begin{aligned} \mathsf {Ch}_{\mathfrak m}(f):=\inf \bigg \{\liminf _{n\rightarrow \infty }\frac{1}{2}\int _X |\nabla f_n|^2d\mathfrak m: f_n\in \mathsf {Lip}_b(X), f_n\rightarrow f \ \mathrm {in} \ L^2(X,\mathfrak m)\bigg \}. \end{aligned}$$

If $\mathsf {Ch}_{\mathfrak m}(f)<\infty$, it was proved in [5, 16] that the set

$$\begin{aligned} {\mathrm G}(f):=\{ g\in L^{2}(X,\mathfrak m)\,:\, \exists (f_{n})_n\subset \mathsf {Lip}_{b}(X), \, f_{n}\rightarrow f, |\nabla f_{n}| \rightharpoonup h\le g \text { in } L^{2}(X,\mathfrak m)\} \end{aligned}$$

is closed and convex, therefore, it admits a unique element of minimal norm called *minimal weak upper gradient* and denoted by $|Df|_{w}.$ The Cheeger energy can be then represented by integration as

$$\begin{aligned} \mathsf {Ch}_{\mathfrak m}(f)= \frac{1}{2}\int _X |D f|_{w}^2d\mathfrak m. \end{aligned}$$

We recall that the minimal weak upper gradient satisfies the following property (see e.g. [6, equation (2.18)]):

19 $$\begin{aligned} |D f|_{w}=0 \ \ \mathfrak m\text {-a.e. on the set} \{f=0\}. \end{aligned}$$

One can show that $\mathsf {Ch}_{\mathfrak m}$ is a 2-homogeneous, lower semicontinuous, convex functional on $L^2(X,\mathfrak m)$ whose proper domain

$$\begin{aligned} \mathbb {V}:=\{f\in L^{2}(X,\mathfrak m)\,:\, \mathsf {Ch}_{\mathfrak m}(f)<\infty \} \end{aligned}$$

is a dense linear subspace of $L^2(X,\mathfrak m)$. It then admits an $L^{2}$ gradient flow which is a continuous semi-group of contractions $(H_{t})_{t\ge 0}$ in $L^{2}(X,\mathfrak m)$, whose continuous trajectories $t \mapsto H_{t} f$, for $f\in L^{2}(X,\mathfrak m)$, are locally Lipschitz curves from $(0,\infty )$ with values into $L^{2}(X,\mathfrak m)$ that satisfy

20 $$\begin{aligned} \frac{d}{d t} H_{t} f \in -\partial \mathsf {Ch}_{\mathfrak m}(H_t f) \ \ \text {for a.e.} \ t\in (0,\infty ). \end{aligned}$$

Here, $\partial$ denotes the subdifferential of convex analysis, namely for every $f\in \mathbb {V}$ we have $\ell \in \partial \mathsf {Ch}_{\mathfrak m}(f)$ if and only if

21 $$\begin{aligned} \int _X \ell (g - f)d \mathfrak m\le \mathsf {Ch}_{\mathfrak m}(g) - \mathsf {Ch}_{\mathfrak m}(f) ,\ \ \ \text {for every} \ g\in L^2(X,\mathfrak m). \end{aligned}$$

We now define the $\mathsf {RCD}(K,\infty )$ condition, introduced and throughly analyzed in [6] (see also [4] for the present simplified axiomatization and the extension to the $\sigma$-finite case).

### Definition 2.2

($\mathsf {RCD}(K,\infty )$ condition) Let $K\in \mathbb {R}$. We say that the metric measure space $(X,\mathsf {d},\mathfrak m)$ is $\mathsf {RCD}(K,\infty )$ if it satisfies the $\mathsf {CD}(K,\infty )$ condition and moreover the Cheeger energy $\mathsf {Ch}_{\mathfrak m}$ is quadratic, i.e. it satisfies the parallelogram identity

22 $$\begin{aligned} \mathsf {Ch}_{\mathfrak m}(f+g)+\mathsf {Ch}_{\mathfrak m}(f-g)=2\mathsf {Ch}_{\mathfrak m}(f)+2\mathsf {Ch}_{\mathfrak m}(g), \quad \forall f,g\in \mathbb {V}. \end{aligned}$$

If $(X,\mathsf {d},\mathfrak m)$ is an $\mathsf {RCD}(K,\infty )$ space, then the Cheeger energy induces the Dirichlet form $\mathcal {E}(f):=2\mathsf {Ch}_{\mathfrak m}(f)$ which is strongly local, symmetric and admits the Carré du Champ

$$\begin{aligned} \Gamma (f):= |Df|_{w}^{2}, \quad \forall f\in \mathbb {V}. \end{aligned}$$

The space $\mathbb {V}$ endowed with the norm $\left\| f \right\| ^2_{\mathbb {V}}:=\left\| f \right\| ^2_{L^2}+\mathcal {E}(f)$ is Hilbert. Moreover, the sub-differential $\partial \mathsf {Ch}_{m}$ is single-valued and coincides with the linear generator $-\Delta$ of the heat flow semi-group $(H_t)_{t\ge 0}$ defined above. In other terms, the semigroup can be equivalently characterized by the fact that for any $f\in L^2(X,\mathfrak m)$ the curve $t\mapsto H_tf\in L^2(X,\mathfrak m)$ is locally Lipschitz from $(0,\infty )$ to $L^2(X,\mathfrak m)$ and satisfies

23 $$\begin{aligned} {\left\{ \begin{array}{ll}\frac{d}{dt} H_tf=\Delta H_tf \ \ \text {for} \ \mathcal {L}^1\text {-a.e } t\in (0,\infty ), \\ \lim _{t\rightarrow 0}H_tf=f, \end{array}\right. } \end{aligned}$$

where the limit is in the strong $L^2(X,\mathfrak m)$-topology.

The semigroup $H_t$ extends uniquely to a strongly continuous semigroup of linear contractions in $L^p(X,\mathfrak m), p\in [1,\infty )$, for which we retain the same notation. Regarding the case $p=\infty$, it was proved in [6, Theorem 6.1] that there exists a version of the semigroup such that $H_tf(x)$ belongs to $\mathcal {C}\cap L^{\infty }((0,\infty )\times X)$ whenever $f\in L^{\infty }(X,\mathfrak m).$ We will implicitly refer to this version of $H_tf$ when *f* is essentially bounded. Moreover, for any $f\in L^2\cap L^{\infty }(X,\mathfrak m)$ and for every $t>0$ we have $H_tf\in \mathbb {V}\cap \mathsf {Lip}(X)$ with the explicit bound (see [6, Theorem 6.5] for a proof)

24 $$\begin{aligned} \left\| |D H_tf|_{w} \right\| _{\infty }\le \sqrt{\frac{K}{e^{2Kt}-1}} \left\| f\right\| _{\infty }. \end{aligned}$$

Two crucial properties of the heat flow are the preservation of mass and the maximum principle (see [5]):

25 $$\begin{aligned} \int _XH_tf \, d\mathfrak m=\int _Xf \, d\mathfrak m, \quad \text {for any } f\in L^1(X,\mathfrak m), \end{aligned}$$

26 $$\begin{aligned} 0\le H_tf\le C, \quad \text {for any } 0\le f\le C\; \mathfrak m\text {-a.e.}, \ C>0. \end{aligned}$$

A result of Savaré [31, Corollary 3.5] ensures that, in the $\mathsf {RCD}(K,\infty )$ setting, for every $f\in \mathbb {V}$ and $\alpha \in [\frac{1}{2},1]$ we have

27 $$\begin{aligned} |D H_tf|_{w}^{2\alpha }\le e^{-2\alpha Kt}H_t\big (|D f|_{w}^{2\alpha }\big ), \quad \mathfrak m\text {-a.e. }. \end{aligned}$$

In particular,

28 $$\begin{aligned} |D H_tf|_{w}\le e^{-Kt}H_t(|D f|_{w}), \quad \mathfrak m\text {-a.e. }. \end{aligned}$$

## Proof of Theorem 1.1

We denote by $I:[0,1]\rightarrow [0,\frac{1}{\sqrt{2\pi }}]$ the Gaussian isoperimetric function defined by $I:=\varphi \circ \Phi ^{-1}$ where

$$\begin{aligned} \Phi (x):=\frac{1}{\sqrt{2\pi }}\int _{-\infty }^x e^{-u^2/2}\, du, \ \ x\in \mathbb {R}, \end{aligned}$$

and $\varphi =\Phi '$. The function *I* is concave, continuous, $I(0)=I(1):=0$ and $0\le I(x) \le I(\frac{1}{2})=\frac{1}{\sqrt{2\pi }},$ for all $x\in [0,1]$. Moreover, $I\in \mathcal {C}^{\infty }((0,1))$, it satisfies the identity

29 $$\begin{aligned} I(x)I''(x)=-1, \quad \text {for every } x\in (0,1). \end{aligned}$$

and (see [10])

30 $$\begin{aligned} \lim _{x \rightarrow 0} \frac{I(x)}{x\sqrt{2\log {\frac{1}{x}}}}=1. \end{aligned}$$

Given $K\in \mathbb {R}$, we define the function $j_K:(0,\infty )\rightarrow (0,\infty )$ as

31 $$\begin{aligned} j_K(t):={\left\{ \begin{array}{ll}\frac{K}{e^{2Kt}-1} \ \ &{}\mathrm {if} \ K\ne 0, \\ \frac{1}{2t} &{}\mathrm {if} \ K=0. \end{array}\right. } \end{aligned}$$

Notice that $j_K$ is increasing as a function of *K*.

The next proposition was proved in the smooth setting by Bakry, Gentil and Ledoux (see [8, 10] and [9, Proposition 8.6.1]).

### Proposition 3.1

(Bakry-Gentil-Ledoux Inequality in $\mathsf {RCD}(K,\infty )$ spaces) Let $(X,\mathsf {d},\mathfrak m)$ be an $\mathsf {RCD}(K,\infty )$ space, for some $K\in \mathbb {R}$. Then, for every function $f\in L^2(X,\mathfrak m)$, $f:X\rightarrow [0,1]$ it holds

32 $$\begin{aligned} |D(H_tf)|_{w}^2\le j_K(t)\Big (\big [I(H_tf)\big ]^2-\big [H_t(I(f))\big ]^2\Big ), \quad \mathfrak m\text {-a.e.},\; \text {for every } t>0. \end{aligned}$$

In particular, for every $f\in L^2\cap L^{\infty }(X,\mathfrak m)$, it holds

33 $$\begin{aligned} \left\| |D(H_tf)|_{w}\right\| _{\infty }\le \sqrt{\frac{2}{\pi }}\sqrt{j_K(t)}\left\| f \right\| _{\infty }, \quad \mathfrak m\text {-a.e.},\; \text {for every } t>0. \end{aligned}$$

### Proof

Given $\varepsilon >0$, $\eta >2\varepsilon$ and $\delta >0$ sufficiently small, consider $f\in L^2(X,\mathfrak m)$ with values in $[0,1-\eta ]$. We define

34 $$\begin{aligned}&\phi _{\varepsilon }(x):=I(x+\varepsilon )-I(\varepsilon ), \end{aligned}$$

35 $$\begin{aligned}&\Psi _{\varepsilon }(s):=\Big [H_s(\phi _{\varepsilon }(H_{t-s}f))\Big ]^2, \quad \text {for every } s\in (0,t). \end{aligned}$$

We notice that $\phi _{\varepsilon }(0)=0$ and $\phi _{\varepsilon }(x)\ge 0$ for every $x\in [0,1-\eta ]$. Moreover, using the property (26), $\phi _{\varepsilon }$ is Lipschitz in the range of $H_{t-s}f$. Since $t\mapsto H_{t}f$ is a locally Lipschitz map with values in $L^p(X,\mathfrak m)$ for $1<p<\infty$ ( [32, Theorem 1, Section III]), we have that $\Psi _{\varepsilon }$ is a locally Lipschitz map with values in $L^1(X,\mathfrak m)$. Let $\psi \in L^1\cap L^{\infty }(X,\mathfrak m)$ be a non-negative function. By the chain rule for locally Lipschitz maps, the fundamental theorem of calculus for the Bochner integral and the properties of the semigroup $H_t$ we have that for any $\varepsilon >0$ and $0<\delta <t$ it holds

36 $$\begin{aligned}&\int _X\bigg (\Big [H_{\delta }(\phi _{\varepsilon }(H_{t-\delta }f))\Big ]^2-\Big [H_{t-\delta }(\phi _{\varepsilon }(H_{\delta }f))\Big ]^2\bigg )\psi \, d\mathfrak m\nonumber \\&\quad =\int _{\delta }^{t-\delta }\bigg (-\frac{d}{ds}\int _X\Big [H_s(\phi _{\varepsilon }(H_{t-s}f))\Big ]^2\psi \, d\mathfrak m\bigg ) ds\nonumber \\&\quad =-2\int _{\delta }^{t-\delta }\bigg (\int _XH_s\big (\phi _{\varepsilon }(H_{t-s}f)\big )H_s\big (\Delta \phi _{\varepsilon }(H_{t-s}f)-\phi _{\varepsilon }'(H_{t-s}f)\Delta H_{t-s}f\big )\psi \, d\mathfrak m\bigg )ds\nonumber \\&\quad =2\int _{\delta }^{t-\delta }\bigg (\int _X H_s\big (\phi _{\varepsilon }(H_{t-s}f)\big )H_s\big (-\phi _{\varepsilon }''(H_{t-s}f)|D H_{t-s}f|_{w}^2\big )\psi \, d\mathfrak m\bigg )ds. \end{aligned}$$

Applying the Cauchy–Schwarz inequality

$$\begin{aligned} H_s(X)H_s(Y)\ge \big [H_s\big (\sqrt{XY}\big )\big ]^2, \end{aligned}$$

and the identity $I(x)I''(x)=-1$, for all $x\in (0,1)$, we get that the right-hand side of (36) is bounded below by

37 $$\begin{aligned} 2\int _{\delta }^{t-\delta }\Bigg (\int _X \Bigg [H_s\Bigg (\sqrt{\bigg (1-\frac{I(\varepsilon )}{I(H_{t-s}f+\varepsilon )}\bigg )|D H_{t-s}f|_{w}^2}\Bigg )\Bigg ]^2\psi \, d\mathfrak m\Bigg )ds. \end{aligned}$$

Noticing that

$$\begin{aligned} \int _X \Bigg [H_s\Bigg (\sqrt{\bigg (1-\frac{I(\varepsilon )}{I(H_{t-s}f+\varepsilon )}\bigg )|D H_{t-s}f|_{w}^2}\Bigg )\Bigg ]^2\psi \, d\mathfrak m\le \int _X \Big [H_s\big (|D H_{t-s}f|_{w}\big )\Big ]^2\psi \, d\mathfrak m\end{aligned}$$

and that, for any fixed $\delta >0$,

$$\begin{aligned} \int _{\delta }^{t-\delta }\bigg (\int _X \Big [H_s\big (|D H_{t-s}f|_{w}\big )\Big ]^2\psi \, d\mathfrak m\bigg )ds <\infty \end{aligned}$$

thanks to the bound (24), we can pass to the limit as $\varepsilon \rightarrow 0$ in (37) using Dominated Convergence Theorem.

Since *I* is continuous, $I(0)=0$ and $I(x)>0$ for every $x\in (0,1)$, using the locality property (19), the Dominated Convergence Theorem yields

38 $$\begin{aligned}&\displaystyle \int _X\bigg (\Big [H_{\delta }(I(H_{t-\delta }f))\Big ]^2-\Big [H_{t-\delta }(I(H_{\delta }f))\Big ]^2\bigg )\psi d\mathfrak m\nonumber \\&\displaystyle \qquad \ge 2\int _{\delta }^{t-\delta }\bigg (\int _X \Big [H_s\big (|D H_{t-s}f|_{w}\big )\Big ]^2\psi d\mathfrak m\bigg )ds, \end{aligned}$$

for every $\delta \in (0,t)$. Now, we can bound the right-hand side of (38) using the inequality (28) to obtain

39 $$\begin{aligned}&\displaystyle 2\int _{\delta }^{t-\delta }\bigg (\int _X \Big [H_s\big (|D H_{t-s}f|_{w}\big )\Big ]^2\psi d\mathfrak m\bigg )ds\nonumber \\&\displaystyle \qquad \ge 2\int _X \bigg (\int _{\delta }^{t-\delta }e^{2Ks}ds\bigg )|D H_tf|_{w}^2\psi \, d\mathfrak m. \end{aligned}$$

From (30) it follows that for every $0<a<1$ there exists $C=C(a)>0$ and ${\bar{x}}={\bar{x}}(a)\in (0,1)$ such that $I(x)\le C x^a$ for all $x\in (0, {\bar{x}})$. In particular, if $g\in L^2(X,\mathfrak m)$, $g:X\rightarrow [0,1-\eta ]$, then $I(g)\in L^p(X,\mathfrak m)$ for every $p>2$. We now apply this argument for $p=4$, so that we can take advantage of the continuity of *I* and the continuity of the semigroup and pass to the limit as $\delta \downarrow 0$. We obtain

40 $$\begin{aligned} \int _X\bigg (\Big [I(H_{t}f)\Big ]^2-\Big [H_{t}(I(f))\Big ]^2\bigg )\psi \, d\mathfrak m\ge \frac{1}{j_K(t)}\int _X |D H_tf|_{w}^2\psi \, d\mathfrak m, \end{aligned}$$

for every $\eta >0$ sufficiently small, every $f\in L^2(X,\mathfrak m)$, $f:X\rightarrow [0,1-\eta ]$.

Now, for $f\in L^2(X,\mathfrak m)$, $f:X\rightarrow [0,1]$, consider the truncation $f_{\eta }:=\min \{f,1- \eta \}$. Applying (40) to $f_{\eta }$, we have

41 $$\begin{aligned} \int _X\bigg (\Big [I(H_{t}f_{\eta })\Big ]^2-\Big [H_{t}(I(f_{\eta }))\Big ]^2\bigg )\psi \, d\mathfrak m\ge \frac{1}{j_K(t)}\int _X |D H_tf_{\eta }|_{w}^2\psi \, d\mathfrak m. \end{aligned}$$

From $f_{\eta }\rightarrow f$ in $L^{2}\cap L^{\infty }(X,\mathfrak m)$ as $\eta \downarrow 0$, we get that $H_tf_{\eta }\rightarrow H_tf$ in $\mathbb {V}$ for every $t>0$; we can then pass to the limit as $\eta \downarrow 0$ in (41) and obtain

$$\begin{aligned} \int _X\bigg (\Big [I(H_{t}f)\Big ]^2-\Big [H_{t}(I(f))\Big ]^2\bigg )\psi \, d\mathfrak m\ge \frac{1}{j_K(t)}\int _X |D H_tf|_{w}^2\psi \, d\mathfrak m. \end{aligned}$$

Since $\psi \in L^1\cap L^{\infty }(X,\mathfrak m)$, $\psi \ge 0$ is arbitrary, the desired estimate (32) follows.

Recalling that $0\le I \le \frac{1}{\sqrt{2 \pi }}$, the inequality (32) yields

42 $$\begin{aligned} |D(H_tf)|_{w}\le \sqrt{\frac{j_K(t)}{2\pi }}, \quad \mathfrak m\text {-a.e.}, \; \text { for every } t>0, \end{aligned}$$

for any $f\in L^2(X,\mathfrak m)$, $f:X\rightarrow [0,1]$. For any $f\in L^2\cap L^{\infty }(X,\mathfrak m)$, write $f=f^{+}-f^{-}$ with $f^{+}=\max \{f, 0\}$, $f^{-}=\max \{-f, 0\}$. Applying (42) to $f^{+}/\Vert f\Vert _{\infty }, f^{-}/\Vert f\Vert _{\infty }$ and summing up we obtain

$$\begin{aligned}&\left\| |D H_tf|_{w}\right\| _{\infty } \le \left\| |D H_tf^{+}|_{w}\right\| _{\infty } + \left\| |D H_tf^{-}|_{w}\right\| _{\infty } \nonumber \\&\qquad \le \sqrt{\frac{2}{\pi }}\sqrt{j_K(t)}\left\| f \right\| _{\infty }, \quad \mathfrak m\text {-a.e., }\forall t>0. \end{aligned}$$

$$\square $$

We next recall the definition of the first non-trivial eigenvalue of the laplacian $-\Delta$. First of all, if $\mathfrak m(X)<\infty$, the non-zero constant functions are in $L^2(X,\mathfrak m)$ and are eigenfunctions of $-\Delta$ with eigenvalue 0. In this case, the first non-trivial eigenvalue is given by $\lambda _{1}$

43 $$\begin{aligned} \lambda _1= \inf \bigg \{\frac{\int _X |D f|_{w}^2 d\mathfrak m}{\int _X |f|^2d\mathfrak m}: \ 0\not \equiv f\in \mathbb {V}, \int _X f d\mathfrak m=0\bigg \}. \end{aligned}$$

When $\mathfrak m(X)=\infty$, 0 may not be an eigenvalue of $-\Delta$ and the first eigenvalue is characterized by

44 $$\begin{aligned} \lambda _0= \inf \bigg \{\frac{\int _X |D f|_{w}^2 d\mathfrak m}{\int _X |f|^2d\mathfrak m}: \ \ 0\not \equiv f\in \mathbb {V} \bigg \}. \end{aligned}$$

Observe that, by the very definition of Cheeger energy (18), the definition (2) of $\lambda _{1}$ (resp. (3) of $\lambda _{0}$) given in the Introduction in terms of slope of Lipschitz functions, is equivalent to (43) (resp. (44)).

It is also convenient to set

45 $$\begin{aligned} J_K(t):=\sqrt{\frac{2}{\pi }}\int _0^t \sqrt{j_K(s)}\, ds, \end{aligned}$$

where $j_{K}$ was defined in (31).

### Proof of Theorem 1.1

**Step 1**: Proof of (9), the case $\mathfrak m(X)=1$.
  First of all, we claim that for any $f\in L^2(X,\mathfrak m)$ with zero mean it holds
  46 $$\begin{aligned} \left\| H_tf\right\| _{2}\le e^{-\lambda _1t}\left\| f\right\| _2. \end{aligned}$$
  To prove (46) let $0\not \equiv f\in L^{2}(X, \mathfrak m)$ such that $0=\int _X fd\mathfrak m=\int _X H_tfd\mathfrak m$. Then
  47 $$\begin{aligned}&2\lambda _1\int _X |H_tf|^2d\mathfrak m\le 2\int _X |D(H_tf)|_{w}^2d\mathfrak m=-2\int _X H_tf\Delta (H_tf)d\mathfrak m\nonumber \\&\qquad =-\frac{d}{dt}\int _X |H_tf|^2d\mathfrak m, \end{aligned}$$
  and the Gronwall’s inequality yields (46).
  Next, we claim that, by duality, the bound (33) implies
  48 $$\begin{aligned} \left\| f-H_t f \right\| _{1}\le J_K(t)\left\| |D f|_{w} \right\| _{1}, \quad \text {for all } f\in \mathsf {Lip}_b(X), \end{aligned}$$
  where $J_{K}(t)$ was defined in (45).
  To prove (48) we take a function *g*, $\left\| g \right\| _{\infty } \le 1$, and observe that
  $$\begin{aligned} \int _X g(f-H_t f)d\mathfrak m&{\,=-}\int _{0}^t\Big (\int _X g\Delta H_s f d\mathfrak m\Big )ds{=}\int _{0}^t \Big (\int _X D H_s g \cdot D f d\mathfrak m\Big )ds \\&\quad \le \left\| |D f|_{w} \right\| _{1}\int _{0}^{t} \left\| |D(H_sg)|_{w}\right\| _{\infty }ds. \end{aligned}$$
  Since *g* is arbitrary, the claimed (48) follows from the last estimate combined with (33).
  We now combine the above claims to conclude the proof. Let $A\subset X$ be a Borel subset and let $f_n\in \mathsf {Lip}_b(X)$, $f_n\rightarrow \chi _A$ in $L^1(X,\mathfrak m)$, be a recovery sequence for the perimeter of the set *A*, i.e.:
  $$\begin{aligned} \mathrm {Per}(A)= \lim _{n\rightarrow \infty } \int _{X} |\nabla f_{n}|\, d\mathfrak m\ge \limsup _{n\rightarrow \infty } \int _{X} |D f_{n}|_{w}\, d\mathfrak m. \end{aligned}$$
  Inequality (48) passes to the limit since $H_t$ is continuous in $L^1(X,\mathfrak m)$ [5, Theorem 4.16] and we can write
  49 $$\begin{aligned} J_K(t)\mathrm {Per}(A)\ge \left\| \chi _A-H_t(\chi _A)\right\| _1=\int _A [1-H_t(\chi _A)]d\mathfrak m+\int _{A^c}H_t(\chi _A)d\mathfrak m\nonumber \\ =2\Big (\mathfrak m(A)-\int _A H_t(\chi _A)d\mathfrak m\Big ) = 2\Big (\mathfrak m(A)-\int _X \chi _A H_{t/2}( H_{t/2} (\chi _A))d\mathfrak m\Big )\nonumber \\ = 2\Big (\mathfrak m(A)-\int _X H_{t/2}(\chi _A) H_{t/2} (\chi _A) d\mathfrak m\Big ) = 2\big (\mathfrak m(A)-\left\| H_{t/2}(\chi _A)\right\| ^2_{2}\big ), \end{aligned}$$
  where we used properties (25), (26), together with the semigroup property and the self-adjointness of the semigroup. We observe that $\int _{X} H_{t/2}(\chi _A-\mathfrak m(A)) \, d\mathfrak m=0$ thanks to (25) and the fact that $H_t \mathbb {1}=\mathbb {1}$ when $\mathfrak m(X)=1$. We can thus apply (46) in order to bound $\left\| H_{t/2}(\chi _A)\right\| ^2_{2}$ in the following way
  50 $$\begin{aligned} \left\| H_{t/2}(\chi _A)\right\| ^2_{2}=\mathfrak m(A)^2+\left\| H_{t/2}(\chi _A-\mathfrak m(A))\right\| ^2_{2}\le \mathfrak m(A)^2+e^{-\lambda _1 t}\left\| \chi _A-\mathfrak m(A)\right\| ^2_{2}. \end{aligned}$$
  A direct computation gives $\left\| \chi _A-\mathfrak m(A)\right\| ^2_{2}=\mathfrak m(A)(1-\mathfrak m(A))$, so that the combination of (49) and (50) yields
  51 $$\begin{aligned} J_K(t)\mathrm {Per}(A)\ge 2\mathfrak m(A)(1-\mathfrak m(A))(1-e^{-\lambda _1t}), \quad \text { for every } t>0. \end{aligned}$$
  Recalling that in the definition of the Cheeger constant *h*(*X*) one considers only Borel subsets $A\subset X$ with $\mathfrak m(A)\le 1/2$, the last inequality (51) gives (9).
**Step 2**: Proof of (10), the case $\mathfrak m(X)=\infty$.
  Arguing as in (47) using Gronwall Lemma, for any $f\in L^2(X,\mathfrak m)$ it holds
  52 $$\begin{aligned} \left\| H_tf\right\| _{2}\le e^{-\lambda _0 t}\left\| f\right\| _2. \end{aligned}$$
  Note that to establish (49), the finiteness of $\mathfrak m(X)$ played no role. Now, we can directly use (52) to bound the right-hand side of the equation (49) in order to achieve
  $$\begin{aligned} \frac{\mathrm {Per}(A)}{\mathfrak m(A)}\ge 2\sup _{t>0}\Big \{\frac{1-e^{-\lambda _0 t}}{J_K(t)}\Big \}, \end{aligned}$$
  for any Borel subset $A\subset X$ with $\mathfrak m(A)<\infty$. The estimate (10) follows.

$$\square $$

### From the implicit to explicit bounds (and sharpness in case $K>0$)

**Proof of Corollary** 1.2 In this section, we show how to derive explicit bounds for $\lambda _1$ (resp. $\lambda _{0}$) in term of the Cheeger constant *h*(*X*), starting from (9) (resp. (10)). We also show that (9) is sharp, since equality is achieved on the Gaussian space. First of all, the expression of the function $J_K$ defined in (45) can be explicitly computed as:

53 $$\begin{aligned} J_K(t)={\left\{ \begin{array}{ll}\sqrt{\frac{2}{\pi K}}\arctan \Big (\sqrt{e^{2Kt}-1}\Big ) \ \ &{}\text {if} \ \ K>0,\\ \frac{2}{\sqrt{\pi }}\sqrt{t} \ \ &{}\text {if} \ \ K=0,\\ \sqrt{-\frac{2}{\pi K}}{{\,\mathrm{arctanh}\,}}{\Big (\sqrt{1-e^{2Kt}}\Big )} \ \ &{}\text {if} \ \ K<0.\end{array}\right. } \qquad \forall t>0 \end{aligned}$$

#### $Case\; K = 0$

When $K=0$, the estimate (9) combined with (53) gives

54 $$\begin{aligned} h(X)\ge \frac{\sqrt{\pi }}{2}\sup _{t>0}\frac{1-e^{-\lambda _1t}}{\sqrt{t}}=\frac{\sqrt{\pi \lambda _1}}{2}\sup _{T>0}\frac{1-e^{-T}}{\sqrt{T}}, \end{aligned}$$

where we set $T=\lambda _{1} t$ in the last identity.

Let $W_{-1}:[-1/e,0)\rightarrow (-\infty ,-1]$ be the lower branch of the Lambert function, i.e. the inverse of the function $x\mapsto xe^x$ in the interval $(-\infty ,-1]$. An easy computation yields

55 $$\begin{aligned}&M:=\sup _{T>0}\frac{1-e^{-T}}{\sqrt{T}}=\frac{\sqrt{-4W_{-1}\Big (-\frac{1}{2\sqrt{e}}\Big )-2}}{2W_{-1}\Big (-\frac{1}{2\sqrt{e}}\Big )}, \nonumber \\&\qquad \mathrm {achieved \ at} \ T=-W_{-1}\Big (-\frac{1}{2\sqrt{e}}\Big )-\frac{1}{2}. \end{aligned}$$

A good lower estimate of *M* is given by $2/\pi$. Using this bound, we obtain

$$\begin{aligned} \lambda _1< \pi h^2(X). \end{aligned}$$

#### $Case\; K\,>\,0$

We start with the following

##### Lemma 3.2

Let $f_1:(0,\infty )\rightarrow (0,\infty )$ be defined as

56 $$\begin{aligned} f_1(x):=\frac{\sqrt{x}}{\arctan \Big (\sqrt{e^{Tx}-1}\Big )}, \end{aligned}$$

where $T>0$ is a fixed number. Then $f_1$ is an increasing function and $f_1(x)\ge \frac{1}{\sqrt{T}}.$

##### Proof

The function $f_1$ is differentiable and the derivative of $f_1$ is non-negative if and only if

$$\begin{aligned} \sqrt{e^{Tx}-1}\arctan \big (\sqrt{e^{Tx}-1}\big )-Tx\ge 0, \ \ \ x>0. \end{aligned}$$

We put $y:=\sqrt{e^{Tx}-1}$ so that we have to prove

57 $$\begin{aligned} y\arctan (y)-\log (y^2+1)\ge 0, \ \ \ y>0. \end{aligned}$$

Called $g_1(y)$ the function $g_1(y):=y\arctan (y)-\log (y^2+1)$, we have that $g_1(0)=0$ and

$$\begin{aligned} g'_1(y)=\arctan (y)-\frac{y}{1+y^2}\ge 0, \end{aligned}$$

so that the inequality (57) is proved and $f_1$ is increasing for any $T>0$. The proof is finished since

$$\begin{aligned} \lim _{x\downarrow 0}f_1(x)=\frac{1}{\sqrt{T}}. \end{aligned}$$

$$\square $$

Rewriting the estimate (9) using (53) in case $K>0$, we obtain

58 $$\begin{aligned} \sqrt{\frac{2}{\pi }}h(X)&\ge \sqrt{K}\sup _{t>0}\frac{1-e^{-\lambda _1t}}{\arctan \Big (\sqrt{e^{2Kt}-1}\Big )} \nonumber \\&=\sqrt{\lambda _1}\sup _{T>0}\frac{\sqrt{\frac{K}{\lambda _1}}}{\arctan \bigg (\sqrt{e^{2\frac{K}{\lambda _1}T}-1}\bigg )}\Big (1-e^{-T}\Big ). \end{aligned}$$

Thanks to the Lemma 3.2 it is clear that we can always obtain the same lower bound of the case $K=0$ (as expected), but this can be improved as soon as we have a positive lower bound of the quotient $K/\lambda _1$. Indeed, let us suppose $K/\lambda _1\ge c>0.$ Then, observing that

$$\begin{aligned} \sup _{T>0} \frac{1-e^{-T}}{\arctan (\sqrt{e^{2cT}-1})}\ge \lim _{T\rightarrow +\infty } \frac{1-e^{-T}}{\arctan (\sqrt{e^{2cT}-1})}=\frac{2}{\pi }, \end{aligned}$$

from (58), we obtain

59 $$\begin{aligned} \sqrt{\frac{2}{c\pi }}h(X)\ge \sqrt{\lambda _1}\sup _{T>0}\frac{1-e^{-T}}{\arctan (\sqrt{e^{2cT}-1})}\ge \frac{2}{\pi } \sqrt{\lambda _1}. \end{aligned}$$

When $X=\mathbb {R}^d$ endowed with the Euclidean distance $\mathsf {d}(x,y)=|x-y|$ and the Gaussian measure $(2\pi )^{-d/2}e^{-|x|^2/2}d\mathcal {L}^d$, $1\le d\in \mathbb {N}$, we have that $h(X)=\sqrt{\frac{2}{\pi }}$, $K=1$ and $\lambda _1=1$ (see [9, Section 4.1]). Thus, we can take $c=1$ and the equality in (59) is achieved, making sharp the lower bound.

#### $Case\; K\,<\,0$

We begin by noticing that

60 $$\begin{aligned} J_K(t)=\sqrt{-\frac{2}{\pi K}}{{\,\mathrm{arctanh}\,}}{\Big (\sqrt{1-e^{2Kt}}\Big )}=\sqrt{-\frac{2}{\pi K}}\log \Big (e^{-Kt}+\sqrt{e^{-2Kt}-1}\Big ). \end{aligned}$$

The following lemma holds:

##### Lemma 3.3

Let $f_2:(0,\infty )\rightarrow (0,\infty )$ be defined as

61 $$\begin{aligned} f_2(x):=\frac{\sqrt{x}}{\log \big (e^{Tx}+\sqrt{e^{2Tx}-1}\big )}, \end{aligned}$$

where $T>0$ is a fixed number. Then, $f_2$ is a decreasing function.

##### Proof

A direct computation shows that the derivative of $f_2$ is non-positive if and only if

$$\begin{aligned} \sqrt{e^{2Tx}-1} \; \log \Big (e^{Tx}+\sqrt{e^{2Tx}-1}\Big )\le 2Txe^{Tx},\quad \text {for all } x>0, \end{aligned}$$

which is equivalent to

62 $$\begin{aligned} \sqrt{1-e^{-2Tx}} \; \log \Big (1+\sqrt{1-e^{-2Tx}}\Big )\le \Big (2-\sqrt{1-e^{-2Tx}}\Big )Tx, \quad \text {for all }x>0. \end{aligned}$$

We put $y:=\sqrt{1-e^{-2Tx}}$, and we write (62) as

$$\begin{aligned} y\log (1+y)+\frac{1}{2}(2-y)\log (1-y^2)\le 0, \quad \text {for all }0<y<1, \end{aligned}$$

which in turn is equivalent to

63 $$\begin{aligned} \left( 1+\frac{y}{2} \right) \log (1+y)+\left( 1-\frac{y}{2}\right) \log (1-y)\le 0, \quad \text {for all }0<y<1. \end{aligned}$$

Now define $g_{2}:(0,1)\rightarrow \mathbb R$ as $g_2(y):=(1+\frac{y}{2})\log (1+y)+(1-\frac{y}{2})\log (1-y)$ and observe that $g_2$ is concave with $g_2(0)=0$, $g'_2(0)=0$. Thus $g_{2}$ is non-positive on (0, 1) and the inequality (63) is proved. $\square$

The combination of (9), (53) and (60) implies that if $(X,\mathsf {d},\mathfrak m)$ is an $\mathsf {RCD}(K,\infty )$ space with $K<0$ and $\mathfrak m(X)=1$ then

64 $$\begin{aligned} h(X)\ge \sqrt{-\frac{\pi K}{2}}\sup _{t>0}\frac{1-e^{-\lambda _1t}}{\log \Big (e^{-Kt}+\sqrt{e^{-2Kt}-1}\Big )}. \end{aligned}$$

We make two different choices:

- When $\lambda _1\le -K$, we choose $t=-\frac{1}{K}$ in (64) so that
  65 $$\begin{aligned} h(X)\ge \sqrt{-\frac{\pi K}{2}}\frac{1-e^{\frac{\lambda _1}{K}}}{\log \Big (e+\sqrt{e^2-1}\Big )} \ge \lambda _1\sqrt{-\frac{\pi }{2K}}\frac{1-\frac{1}{e}}{\log \Big (e+\sqrt{e^2-1}\Big )}, \end{aligned}$$
  where we used the inequality
  $$\begin{aligned} 1-e^{-x}\ge \left( 1-\frac{1}{e} \right) x, \quad \text {for all } 0\le x\le 1. \end{aligned}$$
- When $\lambda _1>-K$, we choose $t=\frac{1}{\lambda _1}$ in (64) so that
  $$\begin{aligned} h(X)\ge \sqrt{\frac{\pi }{2}}\sqrt{\lambda _1}\left( 1-\frac{1}{e}\right) \frac{\sqrt{-\frac{K}{\lambda _1}}}{\log \bigg (e^{-\frac{K}{\lambda _1}}+\sqrt{e^{-2\frac{K}{\lambda _1}}-1}\bigg )}. \end{aligned}$$
  Applying now Lemma 3.3, we obtain
  66 $$\begin{aligned} \lambda _1\le \frac{2\Big (\log \big (e+\sqrt{e^{2}-1}\big )\Big )^2}{\pi \Big (1-\frac{1}{e}\Big )^2}h(X)^2. \end{aligned}$$

The combination of (65) and (66) gives that, if $(X,\mathsf {d},\mathfrak m)$ is an $\mathsf {RCD}(K,\infty )$ space with $K<0$ and $\mathfrak m(X)=1$

67 $$\begin{aligned}&\lambda _1\le \max \bigg \{\sqrt{-K}\frac{\sqrt{2}\log \big (e+\sqrt{e^{2}-1}\big )}{\sqrt{\pi }(1-\frac{1}{e})}h(X),\frac{2\Big (\log \big (e+\sqrt{e^{2}-1}\big )\Big )^2}{\pi \Big (1-\frac{1}{e}\Big )^2}h(X)^2\bigg \}\nonumber \\&\quad <\max \left\{ \frac{21}{10} \sqrt{-K}h(X),\frac{22}{5}h(X)^2 \right\} . \end{aligned}$$

In case $(X,\mathsf {d},\mathfrak m)$ is an $\mathsf {RCD}(K,\infty )$ space with $K<0$ and $\mathfrak m(X)=\infty$ then, using (10) instead of (9), the estimates (64) and (67) hold with $\lambda _{1}$ replaced by $\lambda _{0}$ and *h*(*X*) replaced by *h*(*X*)/2. Thus, in case $\mathfrak m(X)=\infty$, we obtain:

68 $$\begin{aligned}&\lambda _0\le \max \bigg \{\sqrt{-K}\frac{\log \big (e+\sqrt{e^{2}-1}\big )}{\sqrt{2\pi }(1-\frac{1}{e})}h(X),\frac{\Big (\log \big (e+\sqrt{e^{2}-1}\big )\Big )^2}{2\pi \Big (1-\frac{1}{e}\Big )^2}h(X)^2\bigg \}\nonumber \\&\quad <\max \left\{ \frac{21}{20} \sqrt{-K}h(X),\frac{11}{10}h(X)^2 \right\} . \end{aligned}$$

$$\square $$

##### Remark 3.4

Another bound, similar to the one obtained in the case $K>0$, can be achieved in the presence of a lower bound for $K/\lambda _1$, if $\mathfrak m(X)=1$ (resp. a lower bound for $K/\lambda _0$, if $\mathfrak m(X)=\infty$). To see this, let us suppose $K/\lambda _1\ge -c, \ c>0$ (resp. $K/\lambda _0\ge -c$). Then, using (9) (resp. (10)), (53) and Lemma 3.3, we have that (resp. the left-hand side can be improved to $h(X)/\sqrt{2 \pi }$)

69 $$\begin{aligned}&\sqrt{\frac{2}{\pi }}h(X)\ge \sqrt{\lambda _1}\sup _{T>0}\frac{\sqrt{-\frac{K}{\lambda _1}}}{\log \bigg (e^{-\frac{K}{\lambda _1}T}+\sqrt{e^{-2\frac{K}{\lambda _1}T}-1}\bigg )}\Big (1-e^{-T}\Big )\nonumber \\&\quad \ge \sqrt{c\lambda _1}\sup _{T>0}\frac{1-e^{-T}}{\log \big (e^{cT}+\sqrt{e^{2cT}-1}\big )}. \end{aligned}$$

## Appendix A: Cheeger’s Inequality in General Metric Measure Spaces

The Buser-type inequalities of Theorem 1.1 and Corollary 1.2 give an upper bound on $\lambda _{1}$ (resp. on $\lambda _{0}$, in case $\mathfrak m(X)=\infty$) in terms of the Cheeger constant *h*(*X*). It is natural to ask if also a reverse inequality holds, namely if it possible to give a lower bound on $\lambda _{1}$ (resp. on $\lambda _{0}$, in case $\mathfrak m(X)=\infty$) in terms of *h*(*X*). The answer is affirmative in the higher generality of metric measure spaces with a non-negative locally bounded measure *without curvature conditions*, see Theorem 3.6 below. This generalizes to the metric measure setting a celebrated result by Cheeger [17], known as Cheeger’s inequality. In contrast to the previous section, here we do not assume the separability of the space.

A key tool in the proof of Cheeger’s inequality is the co-area formula; more precisely, in the arguments it is enough to have an inequality in the co-area formula. For the reader’s convenience, we give below the statement and a self-contained proof.

### Proposition 3.5

(Coarea inequality) Let $(X,\mathsf {d})$ be a complete metric space and let $\mathfrak m$ be a non-negative Borel measure finite on bounded subsets.

Let $u\in {\mathsf {Lip}}_{b}(X)$, $u:X\rightarrow [0,\infty )$ and set $M=\sup _{X} u$. Then for $\mathcal {L}^{1}$-a.e. $t>0$ the set $\{u> t \}$ has finite perimeter and

70 $$\begin{aligned} \int _{0}^{M} \mathrm {Per}(\{u>t\}) \, d t \le \int _{X} |\nabla u| \, d\mathfrak m. \end{aligned}$$

### Proof

The proof is quite standard, but since we did not find it in the literature stated at this level of generality (typically one assumes some extra condition like measure doubling and gets a stronger statement, namely equality in the co-area formula; see for instance [28]) we add it for the reader’s convenience.

Let $E_{t}:= \{u> t \}$ and set $V(t):=\int _{E_{t}} |\nabla u|\, d\mathfrak m$. The function $t\mapsto V(t)$ is non-increasing and bounded, thus differentiable for $\mathcal {L}^{1}$-a.e. $t>0$.

Since $\int _{X} u \, d\mathfrak m<\infty$, we also have that $\mathfrak m(\{u=t\})=0$ for $\mathcal {L}^{1}$-a.e. $t>0$.

Fix $t>0$ a differentiability point for *V* for which $\mathfrak m(\{u=t\})=0$, and define $\psi :(0,t) \times (0,\infty ) \rightarrow [0,1]$ as

71 $$\begin{aligned} \psi (h,s):= {\left\{ \begin{array}{ll} 0 &{}\quad \text {for } s\le t-h \\ \frac{1}{h}(s-t)+1 &{}\quad \text {for } t-h <s\le t \\ 1 &{} \quad \text {for } s> t. \end{array}\right. } \end{aligned}$$

For $h>0$ define $u_{h}(x)=\psi (h, u(x))$ and observe that the sequence $(u_{h})_{h}\subset {\mathsf {Lip}}_{b}(X)$.

We first claim that

72 $$\begin{aligned} u_{h}\rightarrow \chi _{E_{t}}\quad \text {in } L^{1}(X,\mathfrak m) \quad \text {as } h\downarrow 0. \end{aligned}$$

Indeed

$$\begin{aligned} \int _{X} |u_{h}-\chi _{E_{t}}|\, d\mathfrak m&= \int _{\{ t-h<u\le t\}} \psi (h,u) \, d\mathfrak m\\&\le \mathfrak m\left( \left\{ t-h<u\le t \right\} \right) \rightarrow \mathfrak m(\{u=t\})=0 \quad \text { as } h\downarrow 0, \end{aligned}$$

by Dominated Convergence Theorem, since by assumption *u* has bounded support, $\mathfrak m$ is finite on bounded sets and $\chi _{\{ t-h<u\le t\}} \rightarrow \chi _{\{u=t\}}$ pointwise as $h\downarrow 0$.

In order to prove that $E_{t}$ is a set of finite perimeter it is then sufficient to show that $\limsup _{h\downarrow 0} \int _{X} |\nabla u_{h}|\, d\mathfrak m<\infty$. To this aim observe that

$$\begin{aligned} \int _{X} |\nabla u_{h}|\, d\mathfrak m= \frac{1}{h} \int _{{\{ t-h<u\le t\}}} |\nabla u| \, d\mathfrak m= \frac{V(t-h)-V(t)}{h}. \end{aligned}$$

Since by assumption $t>0$ is a differentiability point for *V*, we obtain that $E_{t}$ is a finite perimeter set satisfying

73 $$\begin{aligned} \mathrm {Per}(E_{t}) \le \lim _{h\downarrow 0} \int _{X} |\nabla u_{h}|\, d\mathfrak m=- V'(t). \end{aligned}$$

Using that (73) holds for $\mathcal {L}^{1}$-a.e. $t>0$ and that *V* is non-increasing, we get

74 $$\begin{aligned} \int _{0}^{M} \mathrm {Per}(E_{t}) \, d t \le - \int _{0}^{M} V'(t) \, d t \le V(0)-V(M)=\int _{X} |\nabla u| \, d\mathfrak m. \end{aligned}$$

$$\square $$

### Theorem 3.6

(Cheeger’s Inequality in metric measure spaces) Let $(X,\mathsf {d})$ be a complete metric space and let $\mathfrak m$ be a non-negative Borel measure finite on bounded subsets.

1. If $$\mathfrak m(X)<\infty $$ then
  75 $$\begin{aligned} \lambda _{1}\ge \frac{1}{4} h(X)^{2}. \end{aligned}$$
2. If $$\mathfrak m(X)=\infty $$ then
  76 $$\begin{aligned} \lambda _{0}\ge \frac{1}{4} h(X)^{2}. \end{aligned}$$

As proved by Buser [11], the constant 1/4 in (75) is optimal in the following sense: for any $h > 0$ and $\varepsilon >0$, there exists a closed (i.e. compact without boundary) two-dimensional Riemannian manifold (*M*, *g*) with $h(M)=h$ and such that $\lambda _{1}\le \frac{1}{4} h(M)^{2}+\varepsilon$.

### Proof

We give a proof of (75), the arguments for showing (76) being analogous (and even simpler).

By the very definition of $\lambda _{1}$ as in (2), for every $\varepsilon >0$ there exists $f\in \mathsf {Lip}_{b}(X)$ with $\int _X f\, d\mathfrak m=0$, $f\not \equiv 0$ such that

77 $$\begin{aligned} \lambda _1\ge \frac{\int _X |\nabla f|^2 \, d\mathfrak m}{\int _X f^2 \, d\mathfrak m} -\varepsilon . \end{aligned}$$

Let *m* be any median of the function *f* and set $f^+:=\max \{f-m,0\}$, $f^-:=-\min \{f-m,0\}$. Applying the co-area inequality (70) to $u=(f^+)^{2}$ (respectively $(f^-)^2$) and recalling the definition of Cheeger’s constant *h*(*X*) as in (4), we obtain

78 $$\begin{aligned}&\int _X |\nabla (f^+)^{2}| \, d\mathfrak m+ \int _X |\nabla (f^-)^{2}| \, d\mathfrak m\nonumber \\&\quad \ge \int _{0}^{\sup \{(f^+)^{2}\}} \mathrm {Per}(\{(f^+)^{2}>t\}) \, d t +\int _{0}^{\sup \{(f^-)^{2}\}} \mathrm {Per}(\{(f^-)^{2}>t\}) \, d t \nonumber \\&\ge h(X) \int _{0}^{\sup \{(f^+)^{2}\}} \mathfrak m(\{(f^+)^{2}>t\}) \, d t +h(X) \int _{0}^{\sup \{(f^-)^{2}\}} \mathfrak m(\{(f^-)^{2}>t\}) \, d t \nonumber \\&=h(X)\int _X (f^+)^{2} d\mathfrak m+h(X) \int _X (f^-)^2 d\mathfrak m= h(X) \int _{X} |f-m|^{2} \, d\mathfrak m. \end{aligned}$$

Since

$$\begin{aligned} |\nabla g^{2}|\le 2 |g| \, |\nabla g|, \end{aligned}$$

and

$$\begin{aligned} |\nabla f^+|\le |\nabla f|, \ \ |\nabla f^-|\le |\nabla f|, \end{aligned}$$

we can apply the Cauchy-Schwarz inequality and get

79 $$\begin{aligned} 2\bigg ( \int _X |\nabla f|^2 \, d\mathfrak m\bigg )^{\frac{1}{2}}\bigg ( \int _X |f-m|^2 \, d\mathfrak m\bigg )^{\frac{1}{2}}\ge \int _X |\nabla (f^+)^{2}| \, d\mathfrak m+ \int _X |\nabla (f^-)^{2}| \, d\mathfrak m, \end{aligned}$$

where we have used that $|f^+| + |f^-|=|f-m|$. It follows from (78) and (79) that for every median *m* of *f* it holds

80 $$\begin{aligned} \frac{\int _X |\nabla f|^2 \, d\mathfrak m}{\int _X |f-m|^2 \, d\mathfrak m}\ge \frac{h(X)^2}{4}. \end{aligned}$$

Finally, since $\int _X f d\mathfrak m=0$ and the mean minimises $\mathbb R\ni c\mapsto \int _X |f-c|^2 d\mathfrak m$, we have

$$\begin{aligned} \frac{\int _X |\nabla f|^2 \, d\mathfrak m}{\int _X |f|^2 \, d\mathfrak m}\ge \frac{\int _X |\nabla f|^2 \, d\mathfrak m}{\int _X |f-m|^2 \, d\mathfrak m} \end{aligned}$$

and we can conclude thanks to (77) and the fact that $\varepsilon >0$ is arbitrary. $\square$
